# Mycoplasmas and Their Antibiotic Resistance: The Problems and Prospects in Controlling Infections

**Published:** 2016

**Authors:** O.A. Chernova, E.S. Medvedeva, A.A. Mouzykantov, N.B. Baranova, V.M. Chernov

**Affiliations:** Kazan Institute of Biochemistry and Biophysics, Kazan Scientific Center, Russian Academy of Sciences, Lobachevskogo Str., 2/31, 420111, Kazan, Russia; Kazan (Volga Region) Federal University, Kremlevskaya Str., 18, 420008, Kazan, Russia

**Keywords:** mycoplasmas, antibiotic resistance mechanisms, omics technologies, bacterial resistome

## Abstract

The present review discusses the problem of controlling mycoplasmas (class Mollicutes), the smallest of self-replicating prokaryotes, parasites of higher eukaryotes, and main contaminants of cell cultures and vaccines. Possible mechanisms for the rapid development of resistance to antimicrobial drugs in mycoplasmas have been analyzed. Omics technologies provide new opportunities for investigating the molecular basis of bacterial adaptation to stress factors and identifying resistomes, the total of all genes and their products contributing to antibiotic resistance in microbes. The data obtained using an integrated approach with post-genomics methods show that antibiotic resistance may be caused by more complex processes than has been believed heretofore. The development of antibiotic resistance in mycoplasmas is associated with essential changes in the genome, proteome, and secretome profiles, which involve many genes and proteins related to fundamental cellular processes and virulence.

## DISCUSSION

Mycoplasmas are of particular interest not only because of the unique organization of these tiny bacteria lacking cell walls, but also for practical considerations. Mycoplasmas are parasites of higher eukaryotes, the causative agents of socially significant infections, and the main contaminants of cell cultures and vaccines. Controlling mycoplasma infections is a serious problem [1-3].

Various mycoplasma inhibition methods have been under development for several decades, but no effective remedies have been discovered [4, 5]. The basic method for inhibiting mycoplasma infections and contamination is based on the administration of antibacterial drugs [2-4]. The rapid development of resistance to antimicrobial agents in mycoplasmas, whose mechanisms are not entirely clear, represents a significant problem. It is believed that the problem of controlling mycoplasma infection and contamination can be cracked by investigating the molecular and genetic adaptation mechanisms of mycoplasmas to stress conditions, which determine the survival of bacteria in various conditions [1-5]. Obviously, such research necessitates the use of an integrated approach involving both conventional and modern methods of analysis of biological material.

In this review, we summarize and analyze data on the mechanisms determining the antibiotic resistance of mycoplasmas. What we know about these mechanisms was largely developed in the period preceding the post-genomic era. Meanwhile, successful implementation of genomic projects and the discovery of omics technologies have led to the development of new approaches in the investigation of the molecular and genetic basis of bacterial adaptation to stress conditions and the discovery of resistomes, the ensemble of all genes and their products involved in the formation of antibiotic resistance in microorganisms [6-13]. This integrated approach produced results indicating that the antibiotic resistance of bacteria may be caused by more complex processes than has previously been thought.

Since Mollicutes class representatives lack cell walls, the main classes of antimicrobial agents, such as betalactam antibiotics, glycopeptides, and fosfomycin, do not affected them. The biological characteristic features of mycoplasmas also result in the ineffectiveness of a number of other substances (sulfonamides, trimethoprim, rifampin, polymyxin, nalidixic acid, linezolid, and some others). Tetracyclines, fluoroquinolones, and macrolides are the most effective anti-mycoplasma agents. They are widely used to suppress mycoplasma infection and contamination of cell cultures. [4] However, recent reports have appeared on a new class of bacteriostatics, deformylase inhibitors, which are active against urogenital mycoplasmosis [5]. However, long-term clinical trials in various regions of the world are required to assess the prospects of these antibiotics.

Antimicrobial peptides (melittin, isolated from bee venom, globomycin, gramicidin C, surfactin, and valinomycin produced by bacteria, alamethicin detected in fungi, A and P1 cecropins, and magainin 2 derived from animal tissues) are not widely used to control mycoplasma at the moment [14-20]. It was found that mycoplasma successfully develops resistance to these drugs [19, 21]. Since data on the mechanisms of mycoplasma resistance to antimicrobial peptides are not yet available, a study of the adaptation of Mollicutes class representatives to antimicrobial agents should focus mainly on the formation of resistance to tetracyclines, fluoroquinolones, and macrolides.

The knowledge about the mechanisms responsible for the resistance of microorganisms to these groups of drugs is based mainly on the results of studies of classical bacteria. This is partly due to the peculiarities of Mollicutes biology, which determine the complexity of their isolation in artificial media and clonal analysis of axenic cultures. The results of a bioinformatics analysis [22-24] are not always consistent with experimental data. Thus, based on an *in silico *analysis of five efflux systems making a substantial contribution to the adaptation of classical bacteria to antibiotics, MATE (the multidrug and toxic compound extrusion family), MFS (the major facilitator superfamily), SMR (the small multidrug resistance family), RND (the resistance-nodulation-cell division superfamily), and ABC (the ATP-binding cassette superfamily) [25, 26], the MATE, MFS, and ABC genes are present in the genomes of some Mollicutes. However, experimental evidence of the contribution of efflux to mycoplasmas antimicrobial resistance has been established only for ABC transporter systems [24, 27, 28].

Either way, the development paths of resistance to tetracyclines, quinolones, and macrolides observed in classic bacteria are largely characteristic of Mollicutes, as well. However, the formation of antimicrobial resistance has different characteristic features in different mycoplasma species. Moreover, even in the case of similar mechanisms, the level of strain sensitivity to the drug can significantly vary *(Table 1)*. Furthermore, the mechanisms that determine antibiotic resistance cannot be identified in some mycoplasma species [5]. This may indicate the existence of as-of-yet undiscovered paths of resistance development in Mollicutes and/or more complex mechanisms of microbial adaptation to antibiotics than was previously thought.

**Table 1:** Resistance to antibiotics (tetracyclines, fluoroquinolones, and macrolides) in mycoplasma associated with target gene mutations [5].

Mycoplasma	Antibiotic class	Resistance		Mutations – positions	MIC range in resistant isolates, μg/ml
		*in vitro*	*in vivo*		
M. pneumoniae	MLSK^a^	+	+	23S rRNA – 2611, 2058, 2059, 2062^b^	64 -> 256 (erythromycin)
	Tetracyclines	+	-	16S rRNA – 968, 1193 (only *in vitro*)	2 (tetracycline)
	Fluoroquinolones	+	-	QRDR^c^ gyrA – 83^d^; gyrB – 426, 447, 466; parC – 78, 80, 84; parE – 439	2–16 (levofloxacin), 8–128 (ciprofloxacin)
M. hominis	MLSK	+	+	23S rRNA – 2610, 2611, 2057, 2059, 2062	16–64 (clindamycin)
	Tetracyclines	+	+	tet(M)-mediated protection of ribosome; 16S rRNA – 346, 965, 966, 967, 1054 (only *in vitro*)	8 -> 64 (tetracycline), 2–8 (tetracycline)
	Fluoroquinolones	+	+	QRDR gyrA – 82, 83, 87, 93; gyrB – 450, 453; parC – 73, 80; parE – 420, 441, 460; Drug efflux (only in vitro, enchances MIC of ciprofloxacin and norfloxacin)	2–32 (levofloxacin), 4–8 (ciprofloxacin)
M. genitalium	MLSK	-	+	23S rRNA – 2058, 2059; ribosomal protein L4	16 -> 64 (erythromycin)
	Tetracyclines	-	-	Resistance genes are not determined	ND^f^
	Fluoroquinolones	-	+	QRDR gyrA – 83, 87, 96; gyrB – 447, 466, parC – 78, 79, 80, 84, 94, 100; parE – 419, 461	ND
Ureaplasma spp.	MLSK	+	+	Ribosomal protein L4; 23S rRNA – 2056, 2057, 2058. Methylation of rRNA by ermB^e^. Drug efflux mediated by msrA, msrB, or msrD products	64 -> 128 (erythromycin)
	Tetracyclines	+	+	tet(M) mediated protection of ribosomes	2 -> 32
	Fluoroquinolones	+	+	QRDR gyrA – 83, 95; gyrB – 119; parC – 80, 84, 123, 134; parE – 151, 249, 274	4–32 (levofloxacin)
M. hyorhinis	MLSK	+	+	23S rRNA – 2059 (in vivo); 23S rRNA – 2059 (in vitro); 23S rRNA – 2597, 2611; 23S rRNA – 2597, 2611	10–100 (tylosin), 25 -> 100 (lincomycin) > 100 (tylosin) 50 (lincomycin) 100 (tylosn), 50 (lincomycin)
	Tetracyclines	-	+	ND	12.5 (chlortetracycline)
	Fluoroquinolones	-	+	ND	1–4 (enrofloxacin)
M. hyopneumoniae	MLSK	-	+	23S rRNA – 2058	> 64 (lincomycin)
	Tetracyclines	+	+	ND	12.5 -≥ 100 (chlortetracycline)
	Fluoroquinolones	+	+	QRDR gyrA – 83; parC (in vivo) – 80, 84, 116	0.25- > 1 (enrofloxacin)
M. bovis	MLSK	+	+	23S rRNA – 748, 2058 (in vitro) 23S rRNA – 748, 752, 2058, 2059 (in vivo); Ribosomal proteins L4 and L22	> 1024 (tylosin), > 256 (tilmicosin) 8–1024 (tylosin), 32 – > 256 (tilmicosin)
	Tetracyclines	+	+	ND	> 32 (oxytetracycline)
	Fluoroquinolones	+	+	QRDR gyrA – 81, 83; parC – 78, 80, 81, 84	2.5–32 (enrofloxacin)
M. gallisepticum	MLSK	+	+	23S rRNA – 2058, 2059 (in vivo); 23S rRNA – 2058, 2503 (in vitro)	0.63–5 (tylosin), 1.25-> 10 (tilmicosin) 256–512 (tilmicosin), 256->512 (erythromycin)
	Tetracyclines	+	+	ND	5 ->16 (oxytetracycline)
	Fluoroquinolones	+	+	QRDR gyrA – 81, 83, 84, 87; gyrB – 426, 464, 465; parC – 64, 80, 81, 84; parE (in vitro) – 420, 463, 467	1–32 (enrofloxacin) 1–10 (enrofloxacin)

^a^MLSK: macrolides, lincosamides, streptogramines, and ketolides.

^b^E. coli numbering system (nucleotide sequence).

^c^QRDR: quinolone resistance determining region.

^d^E. coli numbering system (amino acid sequence).

^e^erm and efflux macrolide genes were found only in one study [29] and were not detected in the others.

^f^ND – not determined.

Tetracyclines are the most widely used agents to control mycoplasma infection of urogenital and respiratory tracts in adults [30, 31]. Additionally, they are frequently used to treat mycoplasma infections in farm animals [5]. The bacteriostatic activity of tetracyclines is based on their capability of reversible binding to the 30S subunit of the bacterial ribosome, inhibition of the interaction between aminoacyl-tRNA and the acceptor site, and thus prevention of the protein synthesis characteristic of these antibiotics [32]. Active cellular efflux of the antibiotic, production of ribosome-protecting proteins (Tet (M), Tet (O), Tet (S), Tet (W), Tet (32), Tet (36), TetB (P), Otr(A), Tet, Tet(Q), and Tet (T)), inhibition of drug influx into the cell, target modification, and antibiotic degradation with enzymes [33, 34] are considered to be *the main mechanisms of tetracyclines resistance in classic bacteria*. Intensive growth of bacterial resistance to tetracyclines is believed to be associated with the active exchange of genes of the key factors involved in the respective processes in bacterial populations [35-38]: the plasmids and mobile genetic elements that are believed to be the main mediators of the horizontal transfer of genetic material.

*The development of tetracycline resistance in mycoplasmas* in some cases is associated with the acquisition of tet(M) determinants located at the Tn916 transposon [39]. The transposon encodes the TetM protein, protecting ribosomes from the effects of tetracyclines. This protein is homologous to the eF-Tu and eF-G elongation factors. It can cause conformational changes in the 30S ribosomal subunit, preventing it from binding to tetracyclines. A high level of tetracycline resistance (MIC ≥ 8 μg/ml) associated with the presence of the tet(M)-determinant causes cross-resistance of mycoplasmas to other tetracycline antibiotics [5, 40]. Furthermore, it is possible that resistance of mycoplasmas to these drugs may be associated with mutations in the tetracycline-binding unit of 16S rRNA [41, 42]. Mycoplasma strains characterized by high tetracycline resistance were also obtained* in vitro *by stepwise selection in media containing gradually increased concentrations of antibiotics [5, 42]. However, the mechanisms of antibiotic resistance could not be determined in these cases.

Macrolide antibiotics are widely used to treat mycoplasmal infections in children (primarily respiratory infections caused by *Mycoplasma pneumonia *and neonatal infections associated with *Ureaplasma spp.), *as well as to suppress mycoplasmoses in animals [5, 43-47]. These antibiotics are often administered in cases where tetracyclines and fluoroquinolones cannot be used.

The antibacterial activity of macrolides is based on the reversible binding of these antibiotics to the 50S ribosomal subunit (including 23S rRNA and some ribosomal proteins, e.g. L4, L22), inducing separation of peptidyl-tRNA from the ribosome, and thus blockage of the synthesis of the peptide chain [48]. *There are three paths of development of macrolide resistance in classical bacteria*: target modification (in particular, structural changes in the 50S ribosomal subunit), change in drug efflux, and enzymatic inactivation of the antibiotic [48, 49].

*Development of macrolide resistance in mycoplasmas* is believed to be associated with inhibition of antibiotic efflux into the cell, as well as structural changes in the 50S ribosomal subunit [5]. In some cases, macrolide resistance in mycoplasmas is associated with changes in the central loop of domain V of 23S rRNA [5, 50]. Mutation in the corresponding gene area leads to increased resistance of certain mycoplasma species to several antibiotics of this group and reduced or lost resistance to others.

Fluoroquinolones are the most popular group of drugs used to inhibit mycoplasma infections and contamination of cell cultures [4, 5, 28]. This is due to the fact that mycoplasma infections often occur in immunodeficient patients and, as a rule, are complex. In such cases, the use of microbicides is recommended. The fluoroquinolone drug ciprofloxacin is a widely used representative of this group [51-53].

The molecular mechanisms of the bactericidal action of fluoroquinolones are based on binding to DNA gyrase and/or DNA topoisomerase IV, which leads to inhibition of bacterial DNA replication [49, 54]. *The main mechanisms of fluoroquinolone resistance of classical bacteria *is associated with target modifications caused by mutations in the QRDR (quinolone resistance-determining region) region of the target genes *gyr*A (DNA gyrase subunit A), *gyr*B (DNA gyrase subunit B), *par*C (topoisomerase IV subunit A), *par*E (topoisomerase IV subunit B), as well as with reduced drug accumulation in the cell (due to active efflux or suppression of influx) and acquired-resistance determinants by horizontal gene transfer [55].

*Development of fluoroquinolone resistance by mycoplasmas* is usually associated with mutations in the QRDR region of the target genes (DNA gyrase and topoisomerase IV). Depending on the antibiotic, significant mutations can occur in the genes of certain enzymes [5]. For example, development of *in vitro *resistance to pefloxacin, ofloxacin, ciprofloxacin, and trovafloxacin in *Mycoplasma hominis *is associated with mutations in the topoisomerase IV gene, while resistance to sparfloxacin occurs due to mutations in the DNA gyrase gene [5, 41, 56]. Fluoroquinolone-resistant clinical isolates of mycoplasma usually demonstrate cross-resistance to all drugs of this group. The resistance level often correlates with the number of mutations and their location [5, 57]. However, a long series of observations of the adaptation to fluoroquinolones in mycoplasmas has shown that displacement of cells lacking the QRDR-mutation from the culture occurs only when bacteria are cultured in media containing high concentrations of ciprofloxacin [58]. With low concentrations of ciprofloxacin, the key role is apparently played by other mechanisms, such as cellular efflux. This type of adaptation to fluoroquinolones, which was identified in a number of bacteria, occurs by means of endogenous ABC-type pumps associated with multidrug resistance (MDR). Increased expression of corresponding genes can determine the MDR-phenotype. ABC-type genes annotated as “suspected MDR genes” were detected in the genomes of certain mycoplasmas [22-4]. According to the results of quantitative competitive RT-PCR, these genes are constitutively expressed in the parental strains, while in the strains with the MDR-phenotype their expression level is increased [18]. However, rapid adaptation of various mycoplasmas to fluoroquinolones still cannot be explained by these factors.

Efforts to figure out the causes of increased fluoroquinolone resistance by microorganisms, which are currently being reported all over the world [56, 59, 60], have led to the assumption that, in addition to these mechanisms, there are other ways that determine the possibility of rapid bacterial adaptation to antibiotics in microbial communities [55]. This assumption is based on the results of both experimental studies and monitoring data in different countries. A very rapid increase in fluoroquinolone resistance is observed in agricultural animals, although these drugs were introduced in veterinary practice only two decades ago [5, 61-63].

Since Mollicutes class representatives are believed to be tachytelic organisms, it is assumed that their rapid adaptation to antimicrobial agents is caused by frequent mutation events, and that changes in the genes of the target proteins are significant [19, 64, 65]. However, according to the results of a complete nucleotide sequence analysis of genes of the *gyrA, gyrB, parC, *and* parE *strains of *Ureaplasma parvum *and *U. urealyticum, a *significant portion of nucleotide substitutions in these mycoplasmic genes represents a specific polymorphism and does not affect antibiotic sensitivity [66]. This finding casts doubt on our knowledge on the mutational mechanisms of antibiotic resistance in mycoplasmas (and other bacteria) and calls for verification of these data using new approaches. Meanwhile, data demonstrating the active role of extracellular vesicles in bacterial adaptation to stress conditions, including antibiotics, have been recently published [3, 67-72]. Vesicles produced by cells contain various compounds and are involved in intercellular interactions in prokaryotes and eukaryotes [69, 73-75]. As early as in 1996, it was established that vesicles of gram-negative bacteria are involved in antibiotic transportation and antibiotic resistance control in bacterial populations [76]. However, the role of vesicles in the bacterial response to antimicrobial agents is only now being extensively studied in connection with the “universality” of vesicular transport, which was esstablished in all organisms, including the smallest prokaryotes, and the development of high-resolution analysis techniques [3, 6-9, 69-71, 73, 74, 76-80].

Active participation of extracellular vesicles in the development of bacterial resistance to fluoroquinolones was first exemplified with *Acholeplasma laidlawii, *mycoplasma infecting humans, animals, plants, and the main contaminant of cell cultures [71, 81]. *A.laidlawii* strains that differed in their susceptibility to ciprofloxacin were obtained by stepwise selection. It was found that vesicles produced by mycoplasma cells growing in a medium with ciprofloxacin mediate the cellular efflux of this drug, have bacteriostatic action against the antibiotic-sensitive *Staphylococcus aureus *strain, and transport the mutant genes of fluoroquinolone target proteins. Differential expression of ABC-transporter genes, which in some bacteria are involved in active efflux of antibiotics and the formation of multi-drug resistance, recorded in response to ciprofloxacin is indicative of the fact that rapid efflux of ciprofloxacin from mycoplasma cells (including through vesicles) can be also associated with modulation of the ABC-transporter system.

Detection of genetic material in vesicles also suggests that they participate in horizontal gene transfer [8, 81-83]. The transport of fluoroquinolone target genes mediated by *A. laidlawii *vesicles may contribute to the rapid expansion of mutant genes in a bacterial population [71, 81]. The possibility of such events is exemplified by *Acinetobacter baumannii. *The extracellular vesicles of this bacterium facilitate the transfer of the *OXA-24 *gene*, *which determines resistance to carbapenems [84]. Thus, transfer of antibiotic resistance factors mediated by the vesicles of certain bacteria may contribute to the survival of various bacteria in a microbial community. An example of such cooperation was illustrated in a *S. aureus *model*, *where a vesiclemediated spread of β-lactamase from these bacteria in microorganism populations resulted in the survival of gram-negative and gram-positive bacteria sensitive to ampicillin on an ampicillin-containing medium [78]. There is clear evidence of the participation of extracellular vesicles in bacterial adaptation to various stress conditions, including antimicrobials. However, it is obvious that comprehensive systematic studies using high-resolution techniques are required in order to uncover the role of vesicular components in the development of bacterial resistance to antibiotics

The development of post-genomic technologies has opened up entirely new possibilities to determine resistomes, the combination of genes and their products involved in the formation of antimicrobial resistance. Information about the resistomes of some bacteria to a number of drugs is now available [85-104]. Such information was recently ,obtained for *A*.*laidlawii *[105]. The information is based on the analysis of complete *A .laidlawii* genomes, as well as the cellular and vesicular proteomes of strains differing in their sensitivity to ciprofloxacin, i.e. the laboratory strain PG8 (MIC 0.5 μg/ml) and the ciprofloxacin-resistant PG8R_10_ strain (MIC 20 μg/ml) derived from the latter by stepwise selection.

**Fig. 1:**
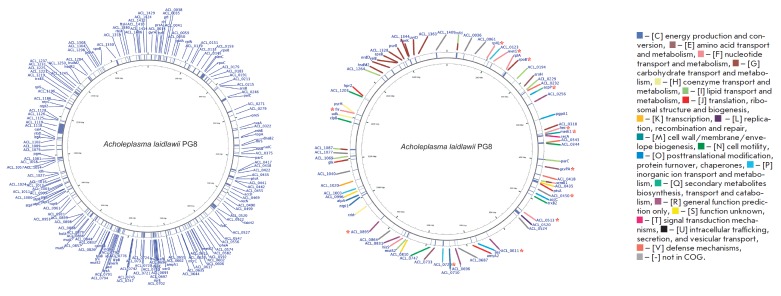
Localization of genes which differ in A.laidlawii PG8 and A.laidlawii PG8R_10_ in their primary structure (A) and genes for proteins differentially expressed in the respective strains (B) on the mycoplasma genome map. * – genes for proteins identified only in the extracellular vesicles derived from A.laidlawii PG8R10. The functional categories were decided according to COG.

A comparative analysis of the nucleotide sequences of *A. laidlawii *and *PG8 *and *A. laidlawii PG8R*10 in the genome of a ciprofloxacin-resistant strain revealed multiple mutations (insertions, deletions, and single nucleotide polymorphism (SNP)) localized in fluoroquinolone target genes (DNA gyrase and DNA topoisomerase), as well as in many other genes whose products participate in various cellular processes and bacterial pathogenicity. A total of 255 mutations in 188 genes were found in the *A. laidlawii PG8R*10 genome (*Fig. 1*). Some of these mutations had been previously identified in other microorganisms in connection with the development of resistance to particular antibiotics (for example, daptomycin resistance in *S. aureus *and multidrug resistance to ciprofloxacin, imipenem, amikacin, minocycline, levofloxacin, piperacillin, tazobactam, ceftazidime, cefotaxime, cefepime, cefoperazone, sulbactam, and meropenem in *A. baumannii* [95, 102]).

A proteomic analysis of *A. laidlawii *PG8 and PG8R10 cells resulted in the identification of proteins whose proportion differed significantly in these strains. A total of 64 such proteins were identified, and only four of them proved to be the products of mutant genes (ACL_0380, ACL_0418, ACL_0435, ACL_0436). Many of these proteins are involved in fundamental cellular processes (energy production, translation, transcription, replication, membrane biogenesis, protein folding, transport and metabolism of amino acids, nucleotides, carbohydrates, lipids, inorganic ions, signal transduction, and defense mechanisms) and bacterial pathogenicity; some of them are involved in the development of antibiotic resistance in other bacteria (for example, to carbapenems in *A. baumannii *and to oxacillin in *S. aureus *[106, 107]).

**Table 2:** Cardiotoxins: properties and conformational characteristics

No	Protein (gene)	NCBI^1^	COG^2^	score^3^	n^4^	%^5^
1	Glycine cleavage system P-protein subunit 1 (ACL_1410)	162447261	E	18	2	12.1
2	Enolase (eno)	162447267	G	662	6	22.7
3	Phosphoglycerate kinase (pgk)	162448052	G	26	2	25.3
4	S-adenosylmethionine synthetase (metK1)	162447194	H	23	2	15
5	50S ribosomal protein L17 (rplQ)	162446985	J	300	2	20.2
6	Methionyl-tRNA synthetase (metG)	162447002	J	19	2	13.4
7	Elongation factor Tu (tuf)	162447058	J	113	3	23.3
8	Methionyl-tRNA formyltransferase (fmt)	162447191	J	17	2	23
9	TrmA family RNA methyltransferase (ACL_0513)	162447375	J	21	2	8.9
10	Ribosome recycling factor (frr)	162447997	J	75	2	40.8
11	DNA-directed RNA polymerase subunit beta (rpoB)	162447041	K	17	2	24.7
12	UDP glucose pyrophosphorylase (galU)	162447697	M	17	2	32.9
13	ABC transporter substrate-binding protein (ACL_0720)	162447580	P	31	2	6.5
14	Acyl carrier protein (acpP)	162447111	Q	131	2	42.1
15	Peptidase U35 (ACL_0611)	162447472	R	47	2	35.4
16	ComEC-like compentence protein (ACL_0895)	162447752	R	295	2	21.2
17	Hypothetical protein (ACL_0450)	162447314	-	22	2	10.5

^1^Protein identification number in the NCBI database.

^2^Proteins classification into functional categories is shown according to COG (E – amino acid transport and metabolism, G – carbohydrate transport and metabolism, H – coenzyme transport and metabolism, J – translation, ribosomal structure and biogenesis, K – transcription, M – cell wall/membrane/envelope biogenesis, P – inorganic ion transport and metabolism, Q – secondary metabolites biosynthesis, transport and catabolism, R – general function prediction only, “-” – not in COG). ■ – bacterial virulence factors

^3^Reliability of protein search in NCBI database using the Mascot software.

^4^the number of various amino acid sequences of peptides which were used to identify the protein.

^5^Percent of amino acid sequence coverage.

We have found significant differences in the proteomic profile of extracellular vesicles in strains that differ in susceptibility to ciprofloxacin (*Table 2*). Thus, 97 proteins were identified in *A. laidlawii* PG8 vesicles and 17 proteins were identified in *A. laidlawii PG8R*10 vesicles; 13 of them are absent in parental strain vesicles [105]. Further, the metallo-β-lactamase protein involved in the hydrolysis of β-lactam antibiotics was found in the vesicles of *A. laidlawii *PG8. Since the action of β-lactam antibiotics is targeted at the bacterial cell wall, which is absent in Mollicutes, the role of this enzyme in *A. laidlawii *PG8 remains unknown. It is possible that *A. laidlawii *PG8*, *similarly to *S. aureus, *may assist other bacteria having cell walls and necessary for the survival of these mycoplasmas in microbiocenosis in adaptation to β-lactam antibiotics [6].

**Fig. 2:**
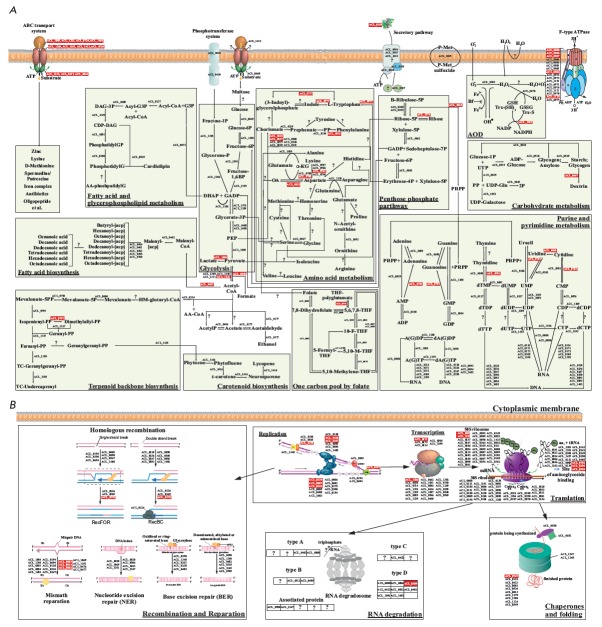
The schemes of metabolic pathways (A) and cellular processes (B) in Acholeplasma laidlawii PG8 (according to [108], NCBI (accession number NC_010163) and KEGG). ■ - products of genes in which non-synonymous SNPs and indels were detected in the case of Acholeplasma laidlawii PG8R_10_. PTS – phosphotransferase system; Fructose-1P – Fructose 1-phosphate; Glucose-6P – Glucose 6-phosphate; Fructose- 6P – Fructose 6-phosphate; Fructose-1,6BP – Fructose 1,6-bisphosphate; DHAP – Dihydroxyacetone phosphate; GA3P – Glyceraldehyde 3-phosphate; 3Pglycerate – glycerate 3-phosphate; PEP – phosphoenolpyruvate; D- Ribulose-5P – D- Ribulose 5-phosphate; Ribose-5P – Ribose 5-phosphate; Xylulose-5P – Xylulose 5-phosphate; Sedoheptulose-7P – Sedoheptulose 7-phosphate; Erythrose-4P – Erythrose 4-phosphate; Glucose-1P – Glucose 1-phosphate; ADP-Gluc – Adenosine diphosphate glucose; UDP-Gluc – Uridine diphosphate glucose; UDP-Gal – Uridine diphosphate galactose; Acetyl-CoA – Acetyl coenzyme A; AcetylP – Acetyl phosphate; Malonyl-CoA – Malonyl coenzyme A; Malonyl-ACP – malonyl:acyl carrier protein; Butyryl-ACP - Butyryl:acyl carrier protein; Hexanoyl -ACP – Hexanoyl:acyl carrier protein; Octanoyl-ACP – Octanoyl:acyl carrier protein; Decanoyl-ACP – Decanoyl:acyl carrier protein; Dodecanoyl-ACP – Dodecanoyl:acyl carrier protein; Tetradecanoyl-ACP – Tetradecanoyl:acyl carrier protein; Hexadecanoyl-ACP – Hexadecanoyl:acyl carrier protein; Octadecanoyl-ACP – Octadecanoyl:acyl carrier protein; G-3P – Glycerol 3-phosphate;Acyl-CoA – Acyl coenzyme A; Acyl-G-3P – Acylglycerol-3-phosphate; DAG-3P – Diacylglycerol- 3-phosphate; CDP-DAG – Cytidinediphosphate-diacylglycerol; Phosphatidyl-GP – Phosphatidylglycerol phosphate; Phosphatidyl-G – Phosphatidylglycerol; AA-CoA – Acetoacetyl coenzyme A; HM-glutaryl-CoA – 3-hydroxy- 3-methylglutaryl-coenzyme A; Mevalonate-5P – Mevalonate-5-phosphate; Mevalonate-5PP – Mevalonate- 5-pyrophosphate; Isopentenyl-PP – Isopentenyl pyrophosphate; Geranyl-PP – Geranyl pyrophosphate; Farnesyl-PP – Farnesyl pyrophosphate; TC-Geranylgeranyl-PP – Di-trans, poly-cis-geranylgeranyl pyrophosphate; TC-undecaprenyl- PP – Di-trans, poly-cis-undecaprenyl-pyrophosphate; Geranylgeranyl-PP – Geranylgeranyl pyrophosphate; 5, 6, 7, 8-THF – 5, 6, 7, 8-tetrahydrofolate; 5,10-M-THF – 5,10-methenyltetrahydrofolate; 10-F-THF – 10-formyltetrahydrofolate; PP – Phenylpyruvate; α-KG – α-Ketoglutaric acid; OA – Oxaloacetate; 5PRPP – 5- Phosphoribosyl pyrophosphate; AMP – Adenosine monophosphate; ADP – Adenosine diphosphate; ATP – Adenosine triphosphate; dADP – Deoxyadenosine diphosphate; dATP – Deoxyadenosine triphosphate; GMP – Guanosine monophosphate; GDP – Guanosine diphosphate; GTP – Guanosine triphosphate; dGDP – Deoxyguanosine diphosphate; dGTP – Deoxyguanosine triphosphate; dTMP – Deoxythymidine monophosphate; dTDP – Deoxythymidine diphosphate; dTDP – Deoxythymidine triphosphate; dUMP – Deoxyuridine monophosphate; dUDP – Deoxyuridine diphosphate; dUTP – Deoxyuridine triphosphate; UMP –Uridine monophosphate; UDP – Uridine diphosphate; UTP – Uridine triphosphate; CMP – Cytidine monophosphate; CDP – Cytidine diphosphate; CTP – Cytidine triphosphate; dCMP – Deoxycytidine monophosphate; dCDP – Deoxycytidine diphosphate; dCTP – Deoxycytidine triphosphate; RNA – Ribonucleic acid; DNA – Deoxyribonucleic acid; mRNA – Messenger ribonucleic acid; tRNA – Transfer ribonucleic acid; A – Adenine; G – Guanine; C – Cytosine; U – Uracil; O2−– Superoxide; H2O2 – Hydrogen peroxide; H2O – Water; GSH – Reduced glutathione; GSSG – Oxidized glutathione; Trx-S2 – Oxidized thioredoxin; Trx-(SH)2 – Reduced thioredoxin; NADPH – Nicotinamide adenine dinucleotide phosphate reduced; NADP – Nicotinamide adenine dinucleotide phosphate; PPi – Pyrophosphate inorganic; Pi – Phosphate inorganic; H+ – Proton; P-Met – Methionine; Fe – Iron.

The contribution of each protein and gene of mycoplasmas, reacting to stress, to the development of ciprofloxacin resistance should be elucidated in the future. However, it is obvious that multiple changes in genomic profiles, as well as the cellular and vesicular proteome, in the ciprofloxacin-resistant *A. laidlawii* strain can determine significant restructuring of biochemical processes in mycoplasma cells (*Fig. 2*). These data were obtained for *Pseudomonas aeruginosa *in connection with the development of resistance to certain antibiotics, including ciprofloxacin [87, 96, 109]. The development of resistance to antimicrobials in various bacterial species proved to be associated with changes not only in the targets of these drugs, but also in many genes and proteins involved in the processes of energy production, transport, and protective mechanisms, as well as in virulence. These results require special attention from researchers involved in the development of control means for pathogenic bacteria and the search for new antimicrobial targets (and virulence factors are possible candidates for this role).

## EXPERIMENTAL

The study of the adaptation of microorganisms to antimicrobial agents using omics technologies is in its infancy. However, the results suggest that the formation of bacterial resistance to antibiotics is, apparently, made possible by more complex mechanisms than has previously been thought. The development of resistance proves to be associated with significant changes in the genomic, transcriptomic, proteomic, and secretomic profiles of microorganisms, which can determine significant restructuring in cellular processes and pathogenicity. Resistome elements that are similar in different bacteria may be indicative of the existence of universal modules regulating cellular reprogramming and ensuring survival in stress conditions. Identification and elucidation of their functional principles is crucial in understanding the “logic of life” of mycoplasma, the rapid bacterial adaptation to stress in microbiocenosis, and finding ways to solve the problem of how to control mycoplasma infection and contamination of cell cultures. Large-scale studies of microorganisms in axenic cultures, as well as in associates in various environments, based on high-tech methodic platforms using meta-omics approaches are required to accumulate the corresponding information.

## Abbreviations

MIC: Minimum Inhibitory Concentration
MLSK: macrolide-lincosamide-streptogramin-ketolide antibiotic group
ABC: ATP-binding cassette
COG: Clusters of Orthologous Groups of Proteins
MATE: Multidrug and Toxic Compound Extrusion family
MDR: Multidrug Resistance
MFS: Major Facilitator Superfamily
SMR: Small Multidrug Resistance family
QRDR: Quinolone Resistance-Determining Region
RND: Resistance-Nodulation-Cell Division superfamily
SNP: Single Nucleotide Polymorphism
